# Significantly Reduced Blood Pressure Measurement Variability for Both Normotensive and Hypertensive Subjects: Effect of Polynomial Curve Fitting of Oscillometric Pulses

**DOI:** 10.1155/2017/5201069

**Published:** 2017-07-12

**Authors:** Fangwei Yang, Fei Chen, Mingping Zhu, Aiqing Chen, Dingchang Zheng

**Affiliations:** ^1^Department of Obstetrics and Gynecology, Yiwu Central Hospital, Yiwu, China; ^2^Department of Electrical and Electronic Engineering, Southern University of Science and Technology, Shenzhen, China; ^3^Health and Wellbeing Academy, Faculty of Medical Science, Anglia Ruskin University, Chelmsford, UK

## Abstract

This study aimed to compare within-subject blood pressure (BP) variabilities from different measurement techniques. Cuff pressures from three repeated BP measurements were obtained from 30 normotensive and 30 hypertensive subjects. Automatic BPs were determined from the pulses with normalised peak amplitude larger than a threshold (0.5 for SBP, 0.7 for DBP, and 1.0 for MAP). They were also determined from cuff pressures associated with the above thresholds on a fitted curve polynomial curve of the oscillometric pulse peaks. Finally, the standard deviation (SD) of three repeats and its coefficient of variability (CV) were compared between the two automatic techniques. For the normotensive group, polynomial curve fitting significantly reduced SD of repeats from 3.6 to 2.5 mmHg for SBP and from 3.7 to 2.1 mmHg for MAP and reduced CV from 3.0% to 2.2% for SBP and from 4.3% to 2.4% for MAP (all *P* < 0.01). For the hypertensive group, SD of repeats decreased from 6.5 to 5.5 mmHg for SBP and from 6.7 to 4.2 mmHg for MAP, and CV decreased from 4.2% to 3.6% for SBP and from 5.8% to 3.8% for MAP (all *P* < 0.05). In conclusion, polynomial curve fitting of oscillometric pulses had the ability to reduce automatic BP measurement variability.

## 1. Introduction

Measurement of blood pressure (BP) is one of the most common and important clinical and diagnostic measurements made by family doctors and hospital physicians. The importance of reliable BP measurement is without doubt, but it is still one of the most poorly performed diagnostic measurements in routine clinical practice [1]. A single BP measurement is often used to determine the diagnosis and treatment (or nontreatment), in spite of high BP variability between measurements. The most noted comment from family doctors and hospital physicians is that consecutive BP measurements vary significantly in the same individual, whether the measurements are taken manually or automatically. It has been reported that BP measurements often vary by more than 10 mmHg between consecutive recordings [2, 3] and that overestimating or underestimating BP by even 5 mmHg can seriously compromise diagnosis and treatment, resulting in the increase of unnecessary costs to healthcare providers [4].

There are two main noninvasive ways of measuring BPs: manual auscultatory method and automatic technique. The gold standard for clinical BP measurement has always been a mercury sphygmomanometer with readings taken by a trained observer using a stethoscope (manual auscultatory technique) [1, 5]. Currently, automatic BP devices have been widely used in many healthcare institutions in place of the manual method or at home by general public for self-measuring BPs to monitor their own health. This is partly because automatic BP devices are very easy to operate. Unfortunately, many users do not strictly follow the BP measurement protocol and guidelines suggested by various clinical hypertension bodies [6, 7]. Some of the BP measurement variability could be reduced by carefully controlling the measurement conditions, including stabilising patient posture, keeping the arm at heart level, using the correct cuff size, and standardising the environment in which BP measurements are made [8, 9]. However, even when these conditions are carefully controlled, BP measurements are still highly variable and accurate determination of the measurement remains inadequate. This concern has been emphasised by the American Heart Association Scientific Statement [1].

The majority of automatic BP devices use the oscillometric technique, where a pressure sensor detects cuff pressure oscillations (or small changes) resulting from the arterial pulse radiating down the arm with each heartbeat. Automatic oscillometric devices analyse the cuff pressure pulse changes (named as oscillometric pulses) to determine systolic and diastolic BPs (SBP and DBP), as well as the mean arterial pressure (MAP) in some devices. Automatic MAP is normally determined from the greatest oscillometric pulses [10, 11]. Different determination algorithms are then developed by adding additional information, such as characteristic ratios of the pulse amplitude to the maximum pulse amplitude, to estimate automatic SBP and DBP [12–14]. During BP measurements, any disturbances that can cause the changes in the oscillometric cuff pressure pulses would result in potential BP measurement variability. Common disturbances that can influence BP measurement variability include heart rate changes, arrhythmias, patient movement, respiratory disturbances, coughing, talking, and muscle tension [3, 8, 15–17]. Therefore, developing a technique which can minimize the measurement variability to achieve reliable BP determination is clinically important, which would significantly aid the effectiveness of automatic devices and provide better clinical data for disease diagnosis.

To achieve reliable automatic BP measurement, modelling approaches, including the polynomial curve fitting to the oscillometric pulses peaks, have been applied on oscillometric pulse peaks to compensate for potential large changes between detectable pulses [14, 18, 19]. However, to the best of our knowledge, there has been no investigation to assess the effect of applying modelling approaches on BP measurement variability. Therefore, the aim of this study was to compare the measurement variabilities from BPs determined by two automatic oscillometric techniques (i.e., with and without polynomial curve fitting) being developed. The BP measurement variability from the two automatic techniques will also be compared with that from the manual auscultatory method.

## 2. Methods

### 2.1. Subjects

International Standards Organization (ISO) requires that the overall mean and standard deviation (SD) of the difference between a new BP measurement technique and the reference BP (from manual auscultatory method) should be ≤5 and 8 mmHg, respectively [20]. Sample size calculation was performed based on a paired *t*-test for mean difference to allow a mean 5 mmHg BP difference to be detected with a typical 8 mmHg SD of BP measurement. 21 subjects were therefore enough to achieve a confidence level of 95% and a statistical power of 80%. 30 healthy normotensive subjects and 30 hypertensive subjects were then recruited to participate in this study. The key subject demographic information including age, sex, height, weight, and arm circumference is summarized in Table 1. The measured SBP was less than 140 mmHg from each of the normotensive subjects and was at least 140 mmHg for the hypertensive subjects. The investigation conformed to the principles in the Declaration of Helsinki. This study received local ethical permission, and all subjects gave their written informed consent to participate in the study.

### 2.2. Blood Pressure Measurement

All the experiments were performed in a quiet clinical measurement room by a trained operator. There were three repeated manual auscultatory measurements for each subject in the sitting position with a time interval of 2 min between each. BP measurements followed the guidelines recommended by the British Hypertension Society (BHS) and European Society of Hypertension (ESH) [6, 7]. The air pressure in the cuff was lineally deflated from approximately 200 mmHg to 30 mmHg at a rate of 2-3 mmHg/s. Manual auscultatory MAP was calculated from the empirical equation: MAP = DBP + (SBP − DBP)/3. There was no significant difference in the manual auscultatory BPs between the three repeated measurements (all *P* > 0.3). The average manual auscultatory BPs from the three repeated measurements were then calculated as the reference BPs for that subject. The overall mean and SD of manual auscultatory BPs from all subjects are also given in Table 1.

**Figure 1:**
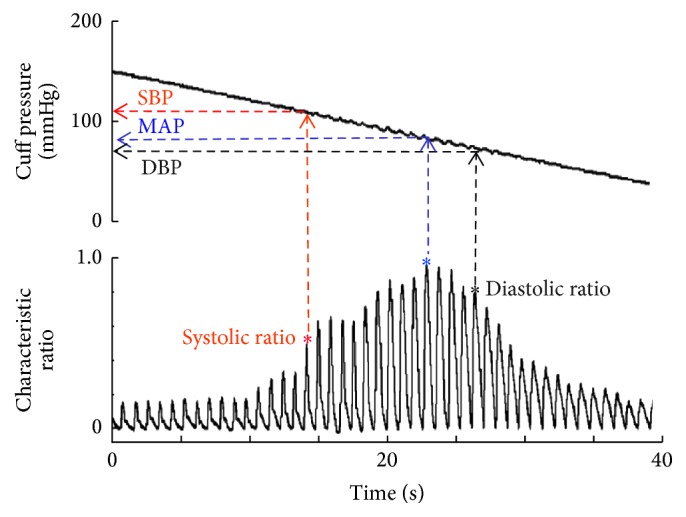
Recorded cuff pressure (top) and extracted oscillometric pulse waveform (bottom) from a subject and illustration of automated BPs (SBP, MAP, and DBP) determination using characteristic ratio information. Oscillometric pulse amplitude is normalised to the maximum pulse peak amplitude to obtain the characteristic ratio. *∗* denotes the oscillometric pulse peaks.

During the manual auscultatory BP measurement, the oscillometric cuff pressure, as shown in Figure 1, was simultaneously recorded with a sampling rate of 2000 Hz for offline analysing oscillometric waveform and determining automated BPs for each subject. In total, 180 oscillometric cuff pressure waveforms were recorded (i.e., from 60 subjects and 3 repeated measurements).

### 2.3. Oscillometric Pulse Extraction and Normalisation

As shown in Figure 1, the oscillometric pulses were extracted from the recorded oscillometric cuff pressure after segmenting each pulse and removing the baseline cuff pressure. The segmentation borders were at the feet of oscillometric pulses, which were manually checked to ensure their accuracy. The peak of each oscillometric pulse was then identified and normalised relative to the maximum oscillometric pulse peak amplitude to obtain the characteristic ratio.

### 2.4. Automatic Oscillometric BP Determination

**Figure 2:**
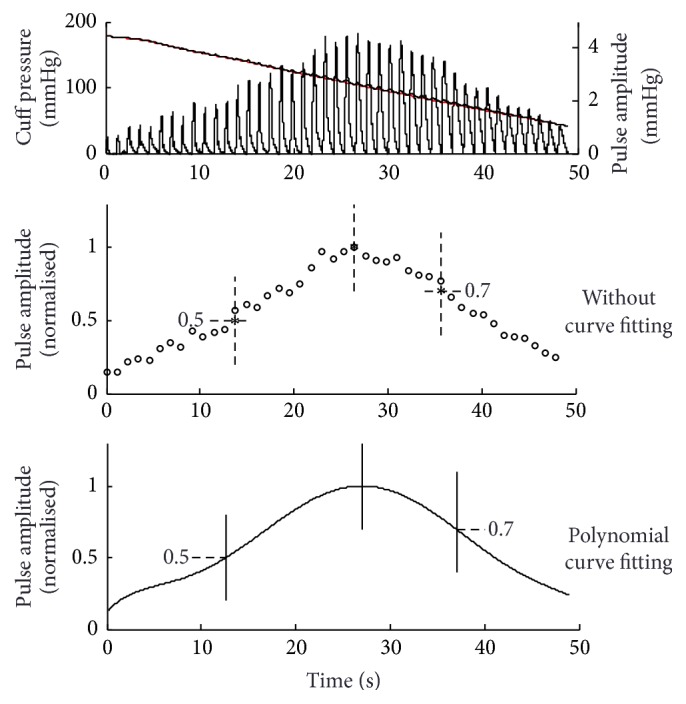
Illustration of the determination of corresponding timings for SBP, DBP, and MAP calculations. Two automatic techniques were used (without polynomial curve fitting: purely relied on the originally recorded oscillometric pulse peak amplitude; with curve fitting: based on the 6th-order polynomial curve fitting of the oscillometric pulse peaks). The thresholds of characteristic ratio of 0.5 and 0.7 are given with horizontal dashed lines. Automated SBP and DBP (without curve fitting) are calculated from the cuff pressures associated with the first and last oscillometric pulses whose normalised amplitude is larger than 0.5 and 0.7, respectively. Automated SBP and DBP (with curve fitting) are calculated from the cuff pressures associated with the thresholds (0.5 and 0.7) crossing the fitted polynomial curve. *∗* denotes the pulses with normalised peak amplitude larger than a threshold (0.5 for SBP, 0.7 for DBP, and 1.0 for MAP).

As shown in Figure 2, for each recorded cuff pressure, two automatic BP determination techniques were developed using the normalised oscillometric pulse peak amplitude information.

The first automatic technique only used the oscillometric pulse peaks data and relied purely on the appearance of the pulse peaks. The automatic SBP and DBP were calculated from the cuff pressures associated with the first and last oscillometric pulses whose normalised amplitude was larger than a threshold. This procedure was equivalent to the principle of manually measuring auscultatory SBP and DBP by listening to the appearance and disappearance of Korotkoff sounds in real clinical measurement. In detail, automatic MAP was obtained from the cuff pressure corresponding to the maximum oscillometric pulse, and automatic SBP and DBP were from the first and last oscillometric pulse peaks above the thresholds of systolic and diastolic characteristic ratios of 0.5 and 0.7, respectively. The fixed thresholds, although arbitrarily selected, were close to the median systolic and diastolic characteristic ratios of 0.48 and 0.71 reported by Amoore et al. and those published by Geddes et al. [12, 21].

The second automatic technique was based on a polynomial curve fitted to the sequence of oscillometric pulse peak amplitudes. In detail, this technique used a 6th-order polynomial curve to fit all available oscillometric pulse peaks. Automatic MAP was then determined from the cuff pressure associated with the peak of the fitted polynomial curve, and automatic SBP and DBP from the cuff pressures associated with the thresholds of 0.5 and 0.7, respectively, crossing the fitted polynomial curve, as shown in Figure 2.

The above two automatic oscillometric techniques used are referred to below simply as automatic techniques with or without curve fitting.

### 2.5. Limits of Agreement between BP Measurement Techniques

The mean and SD of BPs (SBP, DBP, and MAP) were calculated across all subjects, separately for both the normotensive and hypertensive groups and for the three measurement techniques (one manual auscultatory and two automatic oscillometric techniques). The measured BPs from the two automatic oscillometric techniques were compared with the corresponding manual auscultatory readings to obtain the overall mean differences and SDs of their paired differences. Bland-Altman analysis was performed to assess 95% limits of agreement between the automatic oscillometric (for techniques with and without polynomial curve fitting, resp.) and manual auscultatory techniques.

### 2.6. Statistical Analysis of Within-Subject BP Measurement Variability

Within-subject BP measurement variability was calculated from the SD of the three repeated measurements and also expressed as a coefficient of variability (CV = 100 × SD/mean, %), respectively, for the BPs measured from the manual auscultatory and the two automatic oscillometric techniques and separately for both the normotensive and hypertensive groups. Finally, the post hoc multiple comparison in the ANOVA test was performed to compare the two measurement variability parameters (SD of repeats and CV) between the three techniques, separately for the two subject groups. A value of *P* < 0.05 from paired comparison was considered statistically significant.

## 3. Results

### 3.1. Lower Limits of Agreement with Polynomial Curve Fitting

**Figure 3:**
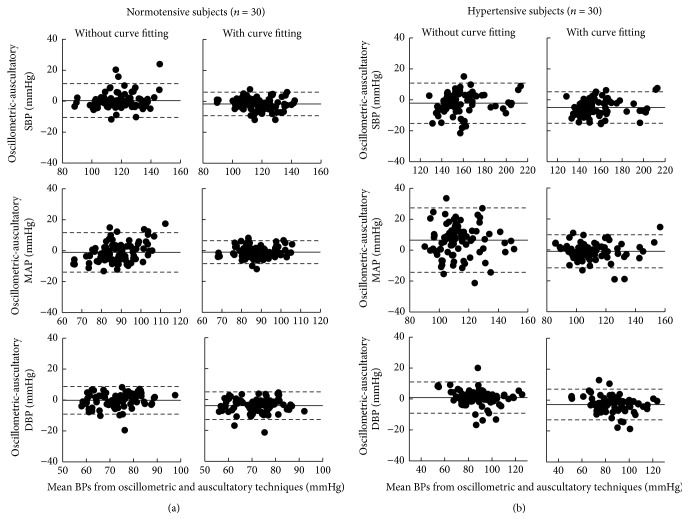
Bland-Altman plots of BP (SBP, DBP, and MAP) differences and 95% limits of agreement (2 SD of paired difference) between the automatic oscillometric (for techniques with and without polynomial curve fitting, resp.) and manual auscultatory methods, separately for the normotensive (a) and hypertensive (b) groups.

The overall means ± SDs of BPs measured from the manual auscultatory technique and the two automatic oscillometric techniques are given in Table 2, separately for the normotensive and hypertensive groups. As shown in Figure 3, in comparison with that without curve fitting, the automatic technique with polynomial curve fitting improved the 95% limits of agreement for the normotensive group from 11.0 to 7.6 mmHg for SBP and from 12.8 to 7.4 mmHg for MAP and for the hypertensive group from 13.0 to 10.2 mmHg for SBP and from 20.8 to 10.8 mmHg for MAP. This reduction of 95% limits of agreement was not observed in DBP from both the normotensive and hypertensive groups.

### 3.2. Reduced Within-Subject Variability with Polynomial Curve Fitting

**Figure 4:**
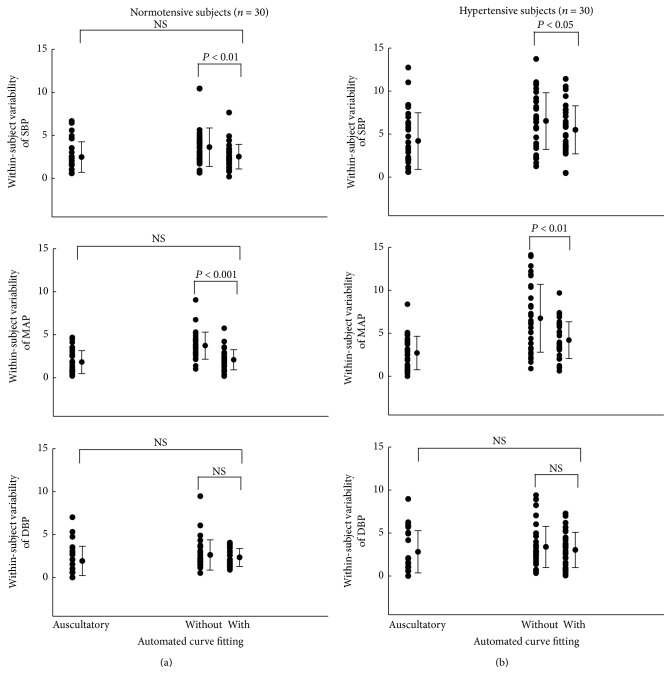
Within-subject variabilities (SD of three repeat measurements in mmHg) of SBP, MAP, and DBP measured from different techniques for both the normotensive (a) and hypertensive (b) groups. In each subfigure, the far left side is from the manual auscultatory technique and the two right ones are from the automatic oscillometric techniques, respectively, without and with polynomial curve fitting. The measurement variability of each individual subject is plotted, together with the mean ± SD of variability from all subjects in that group.

Figure 4 and Table 3 show the within-subject variability parameters (SD of repeats in mmHg and CV in %) for all the BPs (SBP, DBP, and MAP) obtained from the three measurement techniques.

For the normotensive group, the automatic technique with polynomial curve fitting significantly reduced SD of repeats from 3.6 to 2.5 mmHg for SBP and from 3.7 to 2.1 mmHg for MAP (both *P* < 0.01) and the CV from 3.0% to 2.2% for SBP and from 4.3% to 2.4% for MAP (both *P* < 0.01). For the hypertensive group, their corresponding significant changes in SD were from 6.5 to 5.5 mmHg for SBP and from 6.7 to 4.2 mmHg for MAP (both *P* < 0.05) and their corresponding significant changes in CV were from 4.2% to 3.6% for SBP and from 5.8% to 3.8% for MAP (both *P* < 0.05). In terms of the effect on DBP, with the application of polynomial curve fitting, there was no significant reduction of within-subject variability (all *P* > 0.5), separately for both the normotensive and hypertensive groups.

It is also observed that, with the implementation of polynomial curve fitting, the improved within-subject variability approached a level similar to that obtained with the manual auscultatory technique. Especially in the normotensive group, there was no significant difference in SD of repeats and CV for all BPs between the automatic technique with polynomial curve fitting and the manual auscultatory technique (all *P* > 0.1).

## 4. Discussion and Conclusion

This study has quantified the within-subject BP measurement variability, separately for both normotensive and hypertensive subjects. The within-subject BP measurement variability ranged from 1.8 to 3.7 mmHg for normotensive subjects and from 2.7 to 6.7 mmHg for hypertensive subjects, depending on the measurement technique used (i.e., manual auscultatory or automatic oscillometric technique with or without curve fitting). There were still over 40% of normotensive subjects and over 50% of hypertensive subjects having the maximum BP difference (among the three repeats) of more than 4 mmHg. Therefore, as recommended by various BP measurement guidelines [6, 7], at least two BP measurements should be carefully taken with a further repeat measurement if there is an uncertain reading. Our results further suggested that extra care should be given to patients with hypertension to ensure that reliable measurements could be achieved. In future studies, it would be clinically important to comprehensively investigate the possible factors influencing consecutive BP measurements and the reasons accounting for the differences between normotensive and hypertensive groups.

Moreover, this study has shown that the automatic oscillometric technique without curve fitting had relatively larger within-subject BP measurement variability and larger variability of paired BP differences in comparison with the auscultatory technique. It is noted that a recommended slow deflation rate (i.e., 2-3 mmHg/s) was used in this study. With a faster deflation rate in real clinical practice, the measurement variability from the automatic technique without curve fitting could be even larger due to fewer oscillometric peaks detected in a single measurement.

More importantly, this study has shown that, with the implementation of the 6th-order polynomial curve fitting algorithm, the SBP and MAP measurement variabilities have been reduced significantly to the level from the manual auscultatory method. This was observed in both subject groups. The within-subject variability is associated with the smoothness of oscillometric pulse variation [22]. Under stable resting condition, it is expected that there is a smooth decrease in cuff pressure, along with a smooth variation in the oscillometric pulse waveform envelope. With unstable conditions and noisy environment in which BP measurements are made [3, 8, 15–17], as well as the influence of some physiological and biomechanical factors [19, 23], the variability of the oscillometric pulse amplitude changes with deflating cuff pressure becoming large and the smoothness of oscillometric waveform envelope is destroyed. The implementation of polynomial curve fitting on the oscillometric pulse peaks smooths out some random and potential noisy variations in the oscillometric waveform, ensuring a good quality of oscillometric waveform envelope and resulting in reduced within-subject variability.

It is noted that the reduction of measurement variability has not been observed in DBP in this study, suggesting that the variation of the smoothness of oscillometric pulse could change at different cuff pressure regions. For instance, it has been reported that, at the MAP region, the oscillometric waveform envelope is not always a neat bell-shaped curve with a distinct peak [11, 24]. Instead, it may have a plateau, leading to uncertainty of where the maximum peak is. Therefore, the relationship between the underlying causes and the observed variability of the oscillometric pulse amplitude needs to be further investigated.

The fact that the comparison of BP measurement variability between the normotensive and hypertensive groups was not the key objective of this study should be addressed. The analysis and presentation of the results have been performed separately for the two groups in the current study. One of the limitations of this study is that although 30 subjects in each of the normotensive and hypertensive groups were statistically enough for a technology development study, a future clinical population study is recommended with a bigger sample size in large cohorts. It would also be very interesting to understand the association between different physiological and clinical conditions and within-subject measurement variability, which requires some well-designed clinical studies with age-matched subjects and comprehensive clinical information of the subjects. Additionally, the measurement accuracy of automatic oscillometric technique highly depends on the systolic and diastolic characteristic ratios used in the BP determination algorithm. Although the arbitrarily selected thresholds used in this study suit the current study's aim, the measurement accuracy of the automatic technique using polynomial curve fitting principle should be further investigated in a future study with a well-designed clinical validation protocol to obtain the optimal systolic and diastolic characteristic ratios, from where a reliable and accurate BP measurement would produce remarkable progress in diseased diagnosis and early detection of hypertension, generating clinical and societal impacts in healthcare setting.

In conclusion, this study has quantitatively compared the within-subject BP variabilities determined from different measurement techniques and demonstrated that the automatic technique with polynomial curve fitting of oscillometric pulse peaks had the ability to reduce BP measurement variability.

## Figures and Tables

**Table 1:** General data information for the subjects studied.

Subject information	Normotensive group			Hypertensive group		

Number of subjects	30			30		
Number of males	19			20		
Number of females	11			10		

	Min. to max.	Mean	SD	Min. to max.	Mean	SD

Age (years)	22–74	43	19	43–81	69	10
Height (cm)	152–188	171	10	140–193	167	11
Weight (kg)	56–98	71	10	47–138	78	19
Arm circumference (cm)	26–31	28	1.2	23–39	29	3.0
SBP (mmHg)	90–139	117	12	142–207	158	18
DBP (mmHg)	59–89	74	8	50–119	86	15

**Table 2:** Blood pressures measured from different techniques for both normotensive and hypertensive groups and the BP differences between the two automatic oscillometric technique and manual auscultatory techniques (calculated from the automatic-manual auscultatory). The data are presented with means and SDs.

Subject groups	BPs measured from different techniques (mmHg)			Paired difference to manual (mmHg)	
	Manual auscultatory	Automated		Automated	
		Without curve fitting	With curve fitting	Without curve fitting	With curve fitting
Normotensive					
SBP	117.3 ± 12.4	117.6 ± 13.5	115.5 ± 11.9	0.4 ± 5.5	−1.7 ± 3.8
MAP	88.5 ± 8.7	87.4 ± 11.2	87.4 ± 8.8	−1.1 ± 6.4	−1.1 ± 3.7
DBP	74.0 ± 8.1	73.9 ± 9.0	70.0 ± 8.3	−0.2 ± 4.5	−4.0 ± 4.5
Hypertensive					
SBP	158.0 ± 17.6	155.8 ± 18.8	153.0 ± 18.6	−2.2 ± 6.5	−5.0 ± 5.1
MAP	110.2 ± 14.4	116.7 ± 14.8	109.3 ± 14.4	6.5 ± 10.4	−0.9 ± 5.4
DBP	86.2 ± 14.9	87.2 ± 13.9	83.4 ± 13.8	0.9 ± 5.0	−2.9 ± 5.0

**Table 3:** Mean within-subject variabilities of SBP, MAP, and DBP measured from different techniques, separately for both the normotensive and hypertensive groups. The measurement variability was calculated from the SD of the three repeated measurements (in mmHg) and also expressed as a coefficient of variability (CV = 100 × SD/mean, %).

Subject groups	Variability parameters	Auscultatory			Automated-without curve fitting			Automated-with curve fitting		
		SBP	MAP	DBP	SBP	MAP	DBP	SBP	MAP	DBP
Normotensive	SD of repeats	2.5	1.8	1.9	3.6	3.7	2.6	2.5^*∗*^	2.1^*∗*^	2.3
Hypertensive		4.2	2.7	2.8	6.5	6.7	3.4	5.5^#^	4.2^*∗*^	3.0

Normotensive	CV (%)	2.1	2.0	2.6	3.0	4.3	3.5	2.2^*∗*^	2.4^*∗*^	3.3
Hypertensive		2.7	2.5	3.2	4.2	5.8	3.8	3.6^#^	3.8^*∗*^	3.5

*Note*. ^*∗*^*P* < 0.01 and ^#^*P* < 0.05 mean that there is a significant difference in comparison with the automatic technique without polynomial curve fitting.
